# Genotypic Features of Clinical and Bovine *Escherichia coli* O157 Strains Isolated in Countries with Different Associated-Disease Incidences

**DOI:** 10.3390/microorganisms6020036

**Published:** 2018-04-27

**Authors:** Luis Pianciola, Marta Rivas

**Affiliations:** 1 Laboratorio Central, Subsecretaría de Salud de Neuquén, Gregorio Martínez 65, Neuquén 8300, Argentina; 2 Servicio Fisiopatogenia, INEI-ANLIS “Carlos G. Malbrán”, Av. Vélez Sarsfield 563, Buenos Aires 1281, Argentina; mrivas@anlis.gov.ar

**Keywords:** clades, *E. coli* O157, HUS, LSPA-6, Shiga toxin, virulence

## Abstract

There is great geographical variation in the frequency of *Escherichia coli* O157 infections that correlates with important differences in the bovine reservoir of each country. Our group carried out a broad molecular characterization of human and bovine *E. coli* O157 strains circulating in Argentina using different methodologies. Our data allows us to conclude that in Argentina, a high homogeneity is observed in both cattle and human strains, with almost exclusive circulation of strains belonging to the hypervirulent clade 8 described by Manning. The aim of this review was to compare the genetic background of *E. coli* O157 strains isolated in countries that have conducted similar studies, to try to correlate specific O157 genotypes with the incidence and severity of *E. coli* O157 associated diseases. The characteristics of the strains that cause disease in humans reflect the predominant genotypes in cattle in each of the countries analyzed. The main features clearly linked to high incidence or severity of *E. coli* O157 infections are lineage-specific polymorphism assay-6 lineage I/II, clade 8 strains and probably, clade 6 strains, the *stx*_2a_/*stx*_2c_ genotype, the presence of *q*933 and *q*21 simultaneously, and putative virulence factor EC_3286. In countries with an absence of these features in O157 strains, the overall incidence of O157 disease is low. Argentina, where these characteristics are detected in most strains, shows the highest incidence of hemolytic uremic syndrome (HUS) worldwide.

## 1. Introduction

Shiga toxin-producing *Escherichia coli* (STEC) are a heterogeneous group of foodborne pathogens, and *E. coli* O157:H7 is the most common member of this group. The first outbreak associated with this microorganism occurred in Oregon and Michigan, United States (US), in 1982. It was isolated from individuals with bloody diarrhea and severe abdominal cramps who had consumed beef burgers in a well-known food chain [1]. A retrospective search of this serotype in culture collections showed few positive results—only eight strains were deposited before 1982, one in the US, one in the United Kingdom and six in Canada [2]. This low number of O157 strains could be related to a recent emergence of the pathogen and its entry into the agrifood chain.

*E. coli* O157 infections can range from asymptomatic carriage to mild diarrhea, hemorrhagic colitis or hemolytic uremic syndrome (HUS), a severe extraintestinal disease characterized by microangiopathic hemolytic anemia, thrombocytopenia and acute renal failure [3]. Between 3% and 9% of STEC infections progress to HUS [4], and the mortality rate is 3–5% with long-term morbidity occurring in approximately 30% of patients [5]. Enterohemorrhagic *E. coli* (EHEC) is a subgroup of STEC strains characterized as *stx/eae* positive and recognized by their ability to cause severe disease in humans, like HUS. *E. coli* O157:H7 is the most frequent EHEC serotype, but other non-O157 EHEC serogroups are also implicated in HUS [6]. Many countries that use only culture-based confirmation of HUS cases, focusing on sorbitol-nonfermenting strains, may miss non-O157 isolates and therefore, bias the reports on the incidence of each serotype.

Majowicz et al. [7] estimated that STEC causes 2,801,000 acute illnesses and leads to 3890 cases of HUS and 230 deaths annually, worldwide. Important differences exist in the incidence of *E. coli* O157 infections and HUS. Surveillance practices vary considerably, and therefore, caution is required when comparing STEC incidence rates among countries. The incidence of *E. coli* O157 infections per 100,000 inhabitants is approximately 1.0 in the US, 2.1 in England, 0.43 in Germany and 0.08 in France [8]. There are also important regional differences within each country. For example, cases in Scotland increase from west to east and from north to south [9]. In Argentina, where post-diarrheal HUS is endemic, around 400 new cases are reported each year. The disease is the leading cause of acute renal failure in children and the second most frequent cause of chronic renal failure [10]. During 2016, 356 HUS cases were notified to the National Health Surveillance System, which corresponds to a rate of 0.82 cases per 100,000 inhabitants [9]. During the last decade, the annual incidence has ranged from 8 to 12 cases per 100,000 children under 5 years of age [11]. The distribution of cases shows a marked difference between the different regions of the country. The Northern regions show rates below the national average (0.17 and 0.52 per 100,000 inhabitants for northeast and northwest, respectively), the central region is near the national average (0.89 per 100,000), while central Cuyo (1.08 per 100,000) and particularly the Southern region (1.31 per 100,000) have the highest rates in the country.

Ruminants, especially cattle have been recognized as the main reservoir for *E. coli* O157 [12], and many studies have shown large variations in its prevalence in livestock [11]. Sheep, and possibly goats, may be other reservoirs [13]. These animals are not affected by this organism. It can also be found in asymptomatic bisons and cervids, and other mammals, like pigs, camelids, rabbits, horses, dogs and cats. Other free-living wild species, like raccoons, opossums, and rats, may carry this organism in their intestinal tract [14]. *E. coli* O157:H7 may be detected in wild or domesticated birds, including chickens, turkeys, geese, pigeons, starlings, and many other species. Some studies have examined a possible relationship between wild birds and livestock suggesting a role of wild birds in disseminating *E. coli* O157:H7 strains from feedlot pens to the environment [15]. In some instances, it is difficult to prove whether a species is actually a maintenance host or just a temporary carrier [14]. The foods involved are quite variable and include hamburgers, preparations with different types of meat, sausages, dairy products, cider, lettuce, spinach and vegetable sprouts, among others. A study conducted in the UK, Ireland, Denmark, Norway, Finland, the US, Canada and Japan found that the sources of transmission of *E. coli* O157 during outbreaks were different foods (42.2% of cases), dairy products (12.2%), contact with animals (7.8%), water (6.7%), the environment (2.2%), and those of unknown origin (28.9%) [16,17]. Transmission from person-to-person, a process that is especially linked to children’s daycare facilities or nurseries, has also been described. Domestic transmission is more frequent in children under 4 years of age [18].

Genomic studies have allowed researchers to postulate an evolutionary step-by-step model from a non-toxigenic sorbitol fermenter precursor related to the enteropathogen, *E. coli* O55:H7. This ancestor carries the genes of enterocyte effacement, which mediates the intimate attachment of the bacterium to the intestinal epithelium. The first evolutionary steps were the acquisition of the gene coding for Shiga toxin type 2, followed by the somatic antigen switch from O55 to O157 and the acquisition of the large virulence plasmid, pO157. Finally, these strains lost the ability to ferment sorbitol and acquired genes encoding Shiga toxin 1 [19]. Another lineage retained the sorbitol positive phenotype but lost motility, giving rise to the German O157:H- clone [14]. These strains have emerged as important causes of human disease in continental Europe [20]. Bacteriophages have played important roles in the genome changes of *E. coli* O157, and their genes have been gained and lost very dynamically and quickly [21]. The analysis of Single Nucleotide Polymorphisms (SNP) in stable regions of the genome of both ancestral and current strains from different continents and different sources has shown that the strains are very similar. This may be related to a recent origin of *E. coli* O157 [22]. The O157 genome has a 5.5 Mb size and includes a 4.1 Mb backbone shared with most of the *E. coli* serotypes. The rest of the genome originates, largely, from the horizontal transfer of genes, mainly through bacteriophages [23]. The gains and losses of phage genes along with the variation in nucleotides throughout the genome have guided the evolution and diversity of this pathogen [21].

The EDL933 strain associated with the Michigan outbreak and the Sakai City strain were the first *E. coli* O157:H7 genomes to be sequenced [23,24]. At present, a large number of O157:H7 strains have been sequenced, and whole genome comparisons can provide new insight into the underlying epidemiology of this pathogen. In the near future, the application of whole-genome sequencing (WGS) techniques to the analysis of large *E. coli* O157 strain collections will become an invaluable tool for molecular subtyping and will facilitate the establishment of evolutionary relationships [25].

Clinical strains of *E. coli* O157 are characterized by the presence of a specific set of genes and include those coding for Shiga toxins (*stx*_1_, *stx*_2_), intimin (*eae*), and hemolysin (*ehx*A) [26]. There are several subtypes of Stx1 (Stx1a, Stx1c, Stx1d) and Stx2 (Stx2a, Stx2b, Stx2c, Stx2d, Stx2e, Stx2f and Stx2g) [27]. Most human isolates of *E. coli* O157 produce Stx1, Stx2a or Stx2c alone or in combination with other subtypes. Strains that produce Stx2 are more virulent and are more frequently related to severe diseases [28], and those harboring the *stx*_2a_ gene cause more serious illnesses than strains carrying *stx*_2c_.

There is a clear geographical difference in the incidence and severity of infections due to *E. coli* O157. For example, the incidence is generally higher in Scotland than in the rest of European countries [8]. In Latin America, the incidence of HUS is very high in Argentina and lower in the rest of the countries of the region. These differences could be due to (i) the different prevalences of cattle colonization; (ii) the load of *E. coli* O157 in the environment; (iii) the proportion of humans living in areas of high cattle density; (iv) different feeding habits; (v) different genetic structures of pathogen populations; (vi) the pathogen survival in different food types and ecological niches; (vii) differences in genotypes as well as in the infectivity and virulence of circulating strains; or (viii) a combination of these factors [29,30]. The proportion of clinical genotypes in cattle is weakly related to the incidence of HUS in each country, but this is not enough to explain the differences in the international incidence of HUS [24].

A meta-analysis conducted by Salim et al. [31], including 140 studies from 38 countries with more than 220,000 cattle, established a global prevalence of *E. coli* O157 of 5.68% (95% CI, 5.16–6.20). The study showed great regional variation; the highest prevalence was in Africa (31.20%), followed by North America (7.35%), Oceania (6.85%), Europe (5.15%), Asia (4.69%), and the lowest prevalences were detected in Latin America and the Caribbean (1.65%). Large differences were found between the prevalence in feedlot cattle (19.58%, CI 15.57–23.59) and dairy cattle (1.75%, CI 1.26–2.24). Several studies carried out in Argentina have shown prevalences ranging from 0.21% (CI 0.04–0.61) to 4.07% (CI 2.82–5.67) [32,33,34,35,36]. These data show that in Argentina, the country with the highest HUS incidence worldwide, the frequency of cattle colonization with *E. coli* O157 is close to the world’s average and is lower than in many other places with low rates of disease. Therefore, this does not seem to be relevant data to explain the geographical differences in the severity of the associated diseases.

Although cattle and other ruminants are the natural reservoir of *E. coli* O157, only a small subset of serotypes present in animals is related to human diseases [37]. Furthermore, genetic subtypes or lineages of *E. coli* O157 are more associated with human disease, and others are frequent in animals but rare in humans. This could be related to a low virulence or transmissibility to humans of some *E. coli* O157 bovine genotypes [38]. Genes of *E. coli* O157 that encode virulence factors (including products of LEE and pO157) have shown increased expression in clinical genotypes, while genes related to acid resistance and stress fitness were shown to be relatively upregulated in bovine-biased genotypes [39]. Most cattle isolates harbor *stx*_2c_ as the sole gene encoding Stx, whereas *stx*_2a_ is more frequent in patients with severe symptoms [33]. The *E. coli* O157 strains associated with cattle show a pronounced difference in their geographical distribution. This different geographical distribution may have several causes: (a) a different production type or system, like dairy herds or feedlots; (b) age, with a higher prevalence among young animals; (c) season, through an increase in the warmer months of the year; and (d) diet may also affect *E. coli* O157 populations [40,41]. This regional association suggests that strains of *E. coli* O157 have diverged evolutionary in different parts of the world through founder effects or genetic drift or by selective regional pressures. In this way, the difference in the virulence of the strains of each geographical area could explain the differences in the incidence and severity of human diseases related to this microorganism [33,39]. Several researchers have identified genetic markers that are found in different frequencies in strains of clinical cases and animals. Some studies have shown that these genotypic differences are attributable to insertions of bacteriophages, deletions and duplications of DNA fragments of different sizes [42,43]. Initially, an octamer-based genomic scanning was used, through which two lineages were identified: lineage I, composed mainly of strains of clinical origin; and lineage II, composed of strains of animal origin [44]. Subsequently, a new technique was developed, lineage-specific polymorphism assay-6 (LSPA-6), based on the use of a multiplex PCR to detect alleles from six loci that identify lineages I and II [45]. In 2010, Zhang et al. [46] identified another lineage, I/II, with intermediate characteristics between lineages I and II. They also showed that strains from lineage I and I/II produce more Stx2 than strains of lineage II, regardless of their origin. Furthermore, lineage I/II has been related to more severe pathologies, such as HUS [47]. It is interesting to note that the distribution of LSPA-6 lineages in human and cattle isolates is very different in The Netherlands, the US and Japan. A similar pattern occurs when other countries or regions are analyzed.

There is also a great variability in the clinical presentation of pathologies caused by *E. coli* O157. These differences are even more striking when comparing the number of HUS cases and hospitalization rates during different outbreaks. For example, HUS and hospitalization rates during the spinach outbreak in the US in 2006 [48] were higher than those of previous outbreaks in the US [49] and those of the 1996 outbreak in Japan [50]. Manning et al. [51] postulated the existence of *E. coli* O157 strains with great variation in their virulence and suggested that this diversity could explain the different incidences of severe diseases observed during outbreaks. Phylogenetic studies, based on the analysis of SNP in 36 loci of strains from different outbreaks, allowed the description of nine clades. Within them, clade 8 was related to a high number of HUS cases and the highest rates of hospitalization. For that reason, it is known as the hypervirulent clade.

Kulasekara et al. [52] sequenced the complete genome of strain TW14359 related to the spinach outbreak of 2006. The analysis of this sequence and its comparison with the sequences of other *E. coli* O157 strains already sequenced (EDL933, from the US outbreak in 1982 and Sakai, from the 1996 outbreak in Japan) identified some characteristic genetic determinants that could be related to the high virulence of this strain. These putative virulence factors include ECSP_0242, which encodes a factor linked to protein–protein interactions; ECSP_2687, which encodes a protein that reduces the expression of cytokines, decreasing the immune response of the host; ECSP_3620, which encodes the anaerobic nitric oxidase, NorV; ECSP_3286, a protein that binds with high affinity to heme; ECSP_1773, which encodes a protein that interferes with the innate immune response and ECSP_2870/2872, which encodes a protein related to adaptation to plant hosts. The presence of the intact *norV* gene (ECSP_3620) combined with any of the other virulence factors may contribute to the high virulence of these strains.

Although the increased production of Stx2 is a characteristic of clade 8 strains, it is not unique to it and, in addition, not all strains of this clade express high levels of Stx2. The differences in the severity of infections caused by strains of different clades could be explained, at least in part by the differential production of Stx2. In addition, clade 8 strains overexpress LEE genes. Therefore, the virulence of the strains of this clade probably reflects the upregulation of several discrete virulence systems [53]. Several authors have shown that LSPA-6 lineage II strains are less pathogenic, probably due to low Stx production [46,54,55]. Adherence to epithelial cells is higher for clade 8 strains than for clade 2 strains, although no differences have been observed in the invasiveness between the two clades. Strains belonging to clade 8 show upregulation of major virulence genes, including 29 of 41 LEE island genes, which are critical for adherence. The same has been observed for Stx coding genes and for virulence genes encoded in the plasmid, pO157 [56].

The *stx*2 gene is located on the λ family prophages immediately downstream of the phage late promoter (pR’). The expression of the *stx*2 gene is regulated by the transcription of the anti-terminator Q, which initiates the transcription at the late promoter pR’. It has been suggested that the anti-terminator *q* gene on the bacteriophage Q933 could be a useful marker of strains with high toxin production. In contrast, the *q* gene of bacteriophage 21 has been reported from *E. coli* O157:H7 with low Stx production [37,57,58].

STEC strains can colonize cattle for several months, and in this way, may serve as a gene reservoir and may be the origin of *E. coli* O157 genotypes with high virulence. Hence, the importance of characterizing genotypes that circulate in the livestock in a certain area, as the point of origin of strategies to reduce risks to human health [59]. Considering this, our group has previously carried out broad molecular characterization of the human and bovine O157 strains circulating in Argentina using different methodologies (PFGE, LSPA-6, SNP analysis, *stx* subtyping, and putative virulence factors and allele *q* detection, among others). Our data allows us to conclude that in contrast to the great genetic diversity observed in other studies worldwide, in Argentina, high homogeneity is observed in both cattle and human strains, with almost exclusive circulation of strains belonging to the hypervirulent clade 8 described by Manning et al. [51] also carrying also a significant set of putative virulence factors [60,61]. Other methods applied to STEC subtyping, like the Multiple-Locus Variable number tandem repeat Analysis (MLVA) and Multilocus Sequence Typing (MLST), were not used in our previous studies.

The aim of this review was to compare the genetic background of *E. coli* O157 strains isolated in countries that have conducted similar studies to try and correlate specific O157 genotypes with the incidence and severity of *E. coli* O157 associated diseases. This review focuses on *E. coli* O157:H7 (named throughout the manuscript as *E. coli* O157) because this serotype is the etiologic agent of more than 75% of HUS cases in Argentina.

A thorough web-based and PubMed search was conducted to identify relevant studies on these topics. We used the following search terms: *Escherichia coli* O157, *Escherichia coli* O157:H7, *E. coli* O157:H7, *E. coli* O157, STEC, EHEC, VTEC, Shiga toxin combined with LSPA-6 or clades or *Q* alleles or *stx* genotypes or Kulasekara factors or putative virulence factors. 

## 2. Incidence of *E. coli* O157 Infections and HUS in Different Countries

Laboratory surveillance shows that the incidence of *E. coli* O157 infections varies widely from country to country, although these data may be biased. There has been considerable variation in sampling procedures and analysis of specimens, in the methodologies used, and in the epidemiological surveillance systems and records in each country [62]. In Argentina, data on human STEC infections are gathered through different strategies: (i) the reporting of clinical HUS cases to the National Health Surveillance System; these reports, which have been mandatory since 2000, must be immediate and individualized; (ii) the Sentinel Surveillance System through 25 HUS sentinel units; (iii) the laboratory-based surveillance system through the National Diarrheal and Foodborne Pathogens Network; and (iv) the Molecular Surveillance through the PulseNet of Latin America and Caribbean. Data on the incidence of *E. coli* O157 infections and HUS in different countries are shown in Table 1. 

These data show that Argentina, which has the highest incidence of HUS in the world, has low incidence rates of *E. coli* O157 infections. In a previous study, we hypothesized that the strains circulating in this country have a high pathogenic potential to develop HUS, and the clinical evolution would be too fast to be detected at the first stage of diarrhea [76].

## 3. Genetic Features of the Isolates

A collection of 280 strains (226 non-related human and 54 cattle strains) of *E. coli* O157 from Argentina was analyzed by *Xba*I-PFGE, and a great diversity was found [61]. A total of 148 different patterns were detected. The five most common *Xba*I-PFGE patterns identified in both human and animal strains in this study had been included in the National Database. Two of these common patterns, AREXH01.0011 and AREXH01.0022, were predominant in the Argentine Database of *E. coli* O157 in the 1998–2008 period, representing 9.7% and 6.8% of samples, respectively. The AREXH01.0011 pattern is identical to the SMI-H and EXH01.0047 patterns, which are described as predominant in Sweden and the US, respectively [77].

### 3.1. LSPA-6 Analysis

Despite this remarkable diversity observed in PFGE studies, other molecular subtyping techniques applied to study population diversity showed a marked homogeneity of the strains circulating in Argentina. Thus, LSPA-6 showed that 98% of the Argentine strains belong to lineage I/II, which refers to 100% of clinical isolates and all except one strain of the bovine reservoir [61]. This differs from reports from other countries. Frequently, there is greater heterogeneity in the *E. coli* O157 lineages circulating, as well as considerable differences between bovine and human strains. Table 2 shows data from the Argentine strains studied by LSPA-6 compared with data from other countries analyzed with the same methodology.

The predominance of lineage I/II in Argentina is very high in both human and bovine strains. It is noteworthy that strains of lineage II, frequent in the bovine reservoir, were not detected in this collection of strains from Argentina [61]. A similar situation is that of Australia, although this country shows greater heterogeneity in the strains of the bovine reservoir, with a lower frequency of lineage I/II and detection of strains of lineage II that were absent in human strains. Scotland presents a similar situation with a broad predominance of strains of lineage I/II although the other two lineages are also detected in human strains. It is striking that in the bovine reservoir, 100% of the strains belong to lineage I/II. These three countries, in which lineage I/II predominates, present different rates of HUS. Argentina, with the highest incidence of HUS in the world also has the highest frequency of lineage I/II, to which the majority of the strains belong to. A country with a fairly similar situation with respect to LSPA-6 lineage is Australia, although this country has the lowest incidence rate of HUS. Scotland, which has an intermediate incidence rate of HUS, but one of the highest rates in Europe, shows a high prevalence of lineage I/II in human isolates. Belgium, with intermediate values of lineage I/II, is one of the European countries with intermediate incidence of HUS. Other countries, such as the USA, Japan and Canada, in which lineage I/II is not predominant, have lower incidences of HUS. There seems to be an association between the predominance of severe diseases and LSPA I/II lineage, although data from Australia could also indicate that lineage I/II includes strains with different levels of virulence and that in this country, unlike Argentina and Scotland, there are predominately strains with lower virulence in this lineage. A similar situation may be that of The Netherlands. Additional information can be derived from the analysis of data from The Netherlands. While in the bovine reservoir there is a broad predominance of strains of lineage II, they are rare in human cases. This could imply that strains of lineage II represent a lower risk for humans, probably due to a lower level of virulence.

### 3.2. Clade Analysis

In Argentina, an almost exclusive circulation of hypervirulent clade 8 strains is observed, and this could be related to the high incidence of HUS. The clade frequencies in bovine and human *E. coli* O157 strains from different countries are shown in Table 3 and Table 4, respectively.

Countries with intermediate values of clade 8, such as Sweden, also have intermediate rates of HUS. Australia has a very marked predominance of clade 7 strains and is a country with a low incidence of HUS. Something similar happens with Japan, although in this case, there is not such a marked predominance of this clade, and greater heterogeneity is observed in the distribution. Likewise, this country has low HUS rates. The US shows a predominance of isolates belonging to clade 2, although their intermediate HUS incidence could also be related to the intermediate frequency (24.1%) of clade 8 strains. One case to highlight is that of Scotland; although it has low percentages of clade 8 strains (6.8%), it has one of the highest HUS rates in Europe. This country shows a very broad predominance of strains belonging to clades 4/5, 6 or 7, without specifying the particular clade. Probably most of these strains do not belong to clade 7, which, as previously mentioned, includes low virulence strains. Some recent studies have shown a significant relationship between clade 6 strains and the most severe forms of *E. coli* O157 infections [87,88]; this could be the case for Scotland. This point deserves more research. As can be observed in the distribution of clades in bovine strains, especially in Argentina and Australia, the predominance of strains belonging to the same clades in bovine and human populations could demonstrate that most of these last strains have their origin in the bovine reservoir.

### 3.3. stx-Genotype Analysis

Argentina presents a clear predominance of *stx*_2a_/*st*x_2c_ genotypes, which could be directly related to the high incidence of HUS. The distribution of *stx*-genotypes in bovine and human *E. coli* O157 isolated in different countries is shown in Table 4.

In countries with low or intermediate rates of HUS incidence, the *stx*_2a_/*st*x_2c_ genotype is not the predominant one. In countries with a lower incidence of HUS, the *stx*_2c_ variant predominates, in combination with *stx*_1_ (57.0–76.0%) or alone (4.0–30.0%), for example, in Australia. In Japan, the *stx*_1_/*stx*_2a_ genotype clearly predominates (44.0–44.3%). This could confirm a decrease in the virulence of genotype *stx*_2a_ when it is accompanied by *stx*_1_. This last piece of data seems to be ratified by the information from Belgium, since the clear predominance of genotype *stx*_2a_ alone is related to one of the highest HUS rates in Europe. Again, it is observed that the predominance of specific genotypes in humans is related to their distributions in the bovine reservoir. An exception to this is the predominance of the *stx*_2c_ genotype in cattle that is not observed in clinical strains, perhaps related to the lower virulence of this variant.

### 3.4. Anti-terminator Q Alleles Analysis

In relation to the anti-terminator *Q*, the simultaneous presence of the two *q* alleles is related to the higher incidence of HUS, as occurs in the case of Argentina. Table 5 shows the distribution of bovine and human *q* alleles in different countries.

The intermediate incidence of HUS is related to *q*933, which is broadly predominant in Belgium and is present in a similar percentage to *q*21 in The Netherlands. As in the cases analyzed above, the predominance of more virulent genotypes in human strains is linked to a similar predominance in the bovine reservoir. On the other hand, when less virulent forms prevail in cattle, this predominance is lost or diluted in human strains. This could indicate that the less virulent strains have low pathogenic capacity and therefore cause infections in humans less frequently.

### 3.5. Putative Virulence Determinants Analysis

In relation to the putative virulence factors described by Kulasekara et al. [52], the only one that shows an association with high incidences of HUS, such as in Argentina, is ECSP_3286. This factor is related to the extracellular transport of the heme complex. Table 6 shows the distribution of putative virulence factors of Kulasekara in human strains in different countries.

A similar relationship is observed, although to a lesser extent, with factor 2870/2872 which is related to adaptation in plant cells. A case that deserves further analysis is the ECSP_3620 factor, related to the intact gene that encodes a nitric oxide reductase (*nor*V gene). The microorganisms carrying this intact gene have a longer survival time within macrophages since they markedly reduce the level of intracellular nitric oxide. It has also been shown that STEC carriers of this gene produce higher levels of Stx2 within the macrophages. These data could be related to a greater virulence of the *E. coli* O157 that carry this gene [95]. Despite this, the data in Table 6 show that the vast majority of strains are related to a high incidence rate of HUS (Argentina, 95.0–100% of the strains) as well as to lower incidence rates of the disease (Australia, 97.0% of the strains) involving the intact gene. As LSPA-6 lineage I/II predominates in both countries, despite their different incidences of HUS, it could be thought that the presence of ECSP_3620 is a characteristic of this lineage and is not related to the virulence of the strains. This hypothesis was considered in a recent paper by Shimizu et al. [96] where the *nor*V gene was analyzed from the perspective of the evolution of *E. coli* O157.

## 4. Conclusions

The characteristics of the strains that cause disease in humans reflect the predominant genotypes in cattle in each of the countries analyzed. When LSPA-6 lineage II prevails widely in cattle, this predominance is markedly reduced in strains isolated from clinical cases; this is probably related to the low virulence of these strains. The LSPA-6 lineage I/II is related to the most severe cases of *E. coli* O157 infections. Despite this, data from Argentina and Australia show that it is not the only marker of the severity of infections. Clade 8 strains are clearly related to a higher incidence of HUS, although a similar relationship probably exists between strains belonging to clade 6 and serious diseases. The *stx*_2a_/*stx*_2c_ genotype is linked to a high incidence of HUS and the *stx*_2a_ genotype is linked to intermediate incidence. In addition, the *stx*_1a_ gene predominates in strains isolated in countries with a low incidence of HUS. The simultaneous presence of the *q*933 and *q*21 alleles encoding the anti-terminator Q protein is associated with a high incidence of HUS, whereas the presence of *q*933 alone is linked to intermediate incidence. The only putative virulence factor described by Kulasekara that is related to the high incidence of HUS is EC_3286. The presence of the intact *norV* gene may not be related to virulence but could be a marker of LSPA-6 lineage I/II.

## Figures and Tables

**Table 1:** Incidence of *E. coli* O157 infections per 100,000 inhabitants and HUS per 100,000 children under 5 years of age.

Country	*E. coli* O157 Infections	HUS	References
Australia	0.23	0.49	[63,64]
Argentina	0.42	8.8	[11,65]
US	0.97	1.18	[66,67]
Japan	0.71	0.88/10⁵ < 15 years *	[68,69]
The Netherlands	0.46	0.80	[70]
Canada	0.84	1.04	[71,72]
Sweden	0.64	1.19	[70,73]
Belgium	0.36	4.5	[70,74]
Scotland	3.4	3.4	[75]

* No data available for individuals <5 years of age.

**Table 2:** Values (or interval of values from different sources) of lineage-specific polymorphism assay-6 (LSPA-6) lineage frequency in (a) bovine, and (b) human *E. coli* O157 strains from different countries.

(a)				
**Bovine**				
**Country**	**LSPA-LI**	**LSPA-LI/II**	**LSPA-LII**	**References**
Australia	1.0–3.0	81.0–85.0	12.0–18.0	[63,78,79]
Argentina	0–7.0	93.0–100	0	[61,63,79]
US	45.0–63.0	17.0–31.0	3.0–38.0	[78,80]
The Netherlands	1.4	35.6	63.0	[29]
Canada	49.5–75.5	1.5–4.7	20.4–45.8	[81,82]
Scotland	0	100	0	[83]
No data available for Japan, Sweden and Belgium.				
(b)				
**Human**				
**Country**	**LSPA-LI**	**LSPA-LI/II**	**LSPA-LII**	**References**
Australia	4.0–5.0	95.0–96.0	0	[63,78,79]
Argentina	0–4.0	96.0–100	0	[61,63,79]
US	72.0	12.0–14.0	14.0–16.0	[78,79]
Japan	51.1–58.5	30.7–34.4	10.5–14.5	[84,85]
The Netherlands	14.1	77.6	8.2	[29]
Canada	72.8–92.0	4.0–13.6	4.0–15.0	[81,82]
Belgium	10.9	77.2	11.9	[86]
Scotland	1.4	91.9	6.7	[83]
No data available for Sweden.				

**Table 3:** Values (or interval of values from different sources) of clade frequency in (a) bovine and (b) human *E. coli* O157 strains from different countries.

(a)										
**Country**	**Bovine Clades (%)**									**References**
	**1**	**2**	**3**		**4/5**		**6**	**7**	**8**	
Australia	0	0	2		0		23.0	74.0	2.0	[63]
Argentina	0	0	0–8.0		33.3–42.0		0	0	50.0–59.3	[62,63]
The Netherlands	ND	ND	ND		ND		ND	ND	41.1	[29]
Sweden	0	0	0		66.7		0	0	33.3	[89]
Scotland	ND	ND	ND		96.2				3.8	[83]
ND: no data available for US, Japan, Canada and Belgium.										
(b)										
**Country**	**Human Clades (%)**									**References**
	**1**	**2**	**3**	**4/5**		**6**	**7**	**8**	**Other**	
Australia	0	2.0	0	0		6.0	92.0	0	0	[63]
Argentina	0	0	0–3.0	7.2–16.0		0	0	81.0–91.4	0	[60,61,63]
US	0.5	47.4	9.7	5.2		3.2	5.7	24.1	4.2	[51]
Japan	1.5–3.4	9.2–22.9	16.8–34.0	0–1.7		3.9–5.2	16.5–53.8	6.7–12.8	0–12.8	[84,85,87,90]
The Netherlands	ND	ND	ND	ND		ND	ND	38.8	ND	[29]
Sweden	0	1.0	8.0	24.0		12.0	17.0	37.0	1.0	[91]
Scotland	ND	ND	1.4	91.8				6.8	ND	[83]
No data available for Canada and Belgium.										

**Table 4:** Values (or interval of values from different sources) of *stx*-genotype frequencies in (a) bovine *E. coli* O157 strains isolated in different countries.

(a)								
**Country**	**Bovine *stx* Genotypes (%)**							**References**
	**1**	**1/2a**	**1/2a/2c**	**1/2c**	**2a**	**2a/2c**	**2c**	
Australia	0–2.0	1.0–4.0	0	60.0–71.0	0–0.5	0–3.0	25.0–36.1	[63,78,79]
Argentina	0–7.0	4.0–7.4	7.4–10.0	1.9–13.0	9.3–12.0	33.0–55.5	16.6–20.0	[61,63,79]
US	2.1–4.0	44.0–60.0	0–1.4	6.7–21.0	4.0–13.7	0–11.1	6.7–23.8	[78,79,80]
Japan	0–7.0	29.9–30.0	0–0.9	7.0–19.7	9.0–12.0	0–6.8	30.7–47.0	[88,90,91,92]
Sweden	0–1.4	0	0	39.1–46.7	0–2.7	30.4–40.0	13.3–26.4	[86,87,89,90,91]
No data available for The Netherlands, Canada, Belgium and Scotland.								
(b)								
**Country**	**Human *stx* Genotypes (%)**							**References**
	**1**	**1/2a**	**1/2a/2c**	**1/2c**	**2a**	**2a/2c**	**2c**	
Australia	7.6–10.0	3.0–8.0	0	57.0–76.0	0–1.3	0–4.0	4.0–30.0	[63,78,79]
Argentina	0–4.0	0	2.2–10.0	0–8.0	16.0–20.8	53.0–76.1	0.9–8.0	[61,63,79]
US	0–2.8	60.0–63.7	0–1.1	5.0–8.0	17.3–24.0	4.0–4.5	4.0–5.0	[78,79]
Japan	0–0.6	44.0–44.3	0–1.5	4.0–4.9	13.0–16.2	4.0–11.0	21.5–35.0	[87,92]
Belgium	0–3.0	0–5.9	0–2.9	0–35.3	20.6–44.4	8.8–26.0	26.5–27.0	[47,93]
No data available for The Netherlands, Canada, Sweden and Scotland.								

**Table 5:** Values (or range of values from different sources) of *q* allele frequencies in (a) bovine and (b) human *E. coli* O157 strains isolated from different countries.

(a)					
**Country**	**Bovine *q* Alleles (%)**				**References**
	***q*21**	***q*933**	***q*21 + *q*933**	**None**	
Australia	100	0	0	0	[37]
Argentina	24.1	16.7	59.2	0	[61]
US	54.0	44.0	2.0	0	[37]
Japan	82.0	18.0	0	0	[37]
The Netherlands	84.9	9.6	1.4	4.1	[29]
Scotland	25.0	0	75.0	0	[37]
No data available for Canada, Sweden and Belgium.					
(b)					
**Country**	**Human *q* Alleles (%)**				**References**
	***q*21**	***q*933**	***q*21 + *q*933**	**None**	
Argentina	1.8–2.9	15.9	81.2–81.3	0	[60,61]
Japan	19.4	74.6	6.0	0	[94]
The Netherlands	38.8	34.1	23.5	3.5	[29]
Belgium	20.0	61.0	19.0	0	[47]
No data available for Australia, US, Canada, Sweden and Scotland.					

**Table 6:** Values (or range of values from different sources) of putative virulence factors frequency in human *E. coli* O157 strains isolated in different countries.

**Country**	Putative Virulence Factors * in Human Strains (%)						**References**
	**0242**	**1773**	**2687**	**2870/2872**	**3286**	**3620**	
Australia	92.0	23.0	90.0	40.0	2.0	97.0	[78]
Argentina	93.0–100	21.0–33.5	77.0–82.7	63.0–85.7	60.0–88.6	95–100	[1,60,78]
US	35.0	8.0	31.0	28.0	27.0	42.0	[52]

No data available for Japan, The Netherlands, Canada, Sweden, Belgium and Scotland. * Putative virulence factors: 0242 is linked to protein–protein interactions, 1773 encodes a protein that interferes with the immune response, 2687 encodes a protein that reduces the expression of cytokines, 2870/2872 is related to adaptation to plant hosts, 3286 encodes a protein that binds to heme, 3620 encodes anaerobic nitric oxidase.
